# The study of Tau and phospho Tau protein levels in attention deficit and hyperactivity disorder

**DOI:** 10.3906/sag-2012-198

**Published:** 2021-08-30

**Authors:** Hatice SARAÇOĞLU, Eser KILIÇ, Esra DEMİRCİ

**Affiliations:** 1 Department of Medical Biochemistry, Faculty of Medicine, Erciyes University, Kayseri Turkey; 2 Department of Child and Adolescent Psychiatry, Faculty of Medicine, Erciyes University, Kayseri Turkey

**Keywords:** Attention deficit and hyperactivity disorder, Tau, phospho Tau

## Abstract

**Background/aim:**

Attention deficit and hyperactivity disorder (ADHD) is a widespread neurodevelopmental disorder that begins in childhood and has negative consequences throughout adult life. The etiology and pathogenesis of ADHD are still unclear. Tau protein is a soluble microtubule-related protein expressed by neurons and localized in the cytoplasm as well as axons. Tau protein provides stability of microtubule in two ways: phosphorylation and isoforms. The excessive phosphorylation of Tau separates the protein from the microtubule, thus making it unstable. In this study, we aimed to investigate whether there is a relationship between serum Tau protein and phospho Tau** (**p-Tau_181_) levels and ADHD occurrence.

**Materials and methods:**

This study included 26 male children aged 7–12 years with newly diagnosed ADHD, who had previously not used any medication for ADHD, and 26 male healthy children. Serum Tau and p-Tau181 concentrations were performed by enzyme-linked immunosorbent assay (ELISA).

**Results:**

In patients, the Tau levels were not significantly different from those of the controls; the p-Tau181 levels were significantly higher than those of the controls.

**Conclusion:**

We concluded that high p-Tau181 might be associated with the progression of ADHD and cognitive changes in ADHD.

## 1. Introduction

Attention deficit and hyperactivity disorder (ADHD) is one of the common neuropsychiatric disorders of childhood. It is a lifelong disorder that causes significant loss of functionality in many areas. It negatively affects the quality of life of the patient and family, and therefore the society [1,2]. ADHD is defined as the inability to sustain attention and/or symptoms of hyperactivity and impulsivity that are more severe, continuous, or frequent compared to individuals of similar age and developmental levels [3].

Neuropsychiatric disorders, such as ADHD, are quite common in the population. Although they may stand to express modifications in brain duty, they are not defined by clear neuropathology and the underlying biochemical conditions are mainly unrecognized. In other words, the etiology and pathogenesis of ADHD are still unclear [4]. Therefore, new research to explore ADHD is presently ongoing. 

Tau is a soluble microtubule-associated protein required for polymerization and stabilization of the microtubular cytoskeleton [5]. It is placed in the brain and spinal cord, primarily in axons. Tau protein undergoes many posttranslational modifications, and the most important is phosphorylation, which regulates both normal and pathological functions of Tau. Over phosphorylation reduces Tau (p-Tau) affinity for microtubules, damaging normal functions and decreasing biological activity [6]. p-Tau separates from the microtubule and thus destabilizes it [7]. The neuron loses viability as a result of the cytoskeleton and cellular transport disruption. Tau is released into the cerebrospinal fluid (CSF) and plasma by disrupting cell integrity. Therefore, CSF and plasma Tau/pTau levels can be used as a marker in the diagnosis of some diseases and in determining the severity of axonal damage [8].

There are few studies on psychiatric disorders related to Tau and pTau pathology. In a study, serum total Tau and pTau levels were found to be low in schizophrenia [9]. Studies in rats showed that chronic stress triggers Tau hyperphosphorylation [10]. In another study, autopsy samples were examined and Tau was shown to be higher in suicide cases [11]. Total Tau levels were found to be significantly lower in children with autism spectrum disorder compared to the control [12]. These findings suggest that Tau and pTau may also play a role in the pathogenesis of psychiatric disorders. Information on serum Tau and phospho Tau (p-Tau181) protein levels in ADHD and its role in the pathogenesis of the disorder is very weak. There is a study in the literature on ADHD, and only serum Tau levels were evaluated, not pTau, and they were found to be high in the patient group [13]. Differently, in the present study, we aimed to evaluate both serum total Tau and p-Tau181 levels in ADHD.

## 2. Materials and methods

### 2.1. Type of population

This study was performed in the Biochemistry and Child and Adolescent Psychiatry departments of Erciyes University Medical Faculty. Twenty-six male children aged 7–12 years, with newly diagnosed ADHD, who had formerly not taken any medication for ADHD were included. They were examined at the Child and Adolescent Psychiatry Department, between August 2017 and July 2018. ADHD was diagnosed by two child and adolescent psychiatrists based on the DSM V-TR diagnostic criteria and the short form of the Conners’ Parent Rating Scale.  Twenty-six healthy male children aged 7–12 years who applied to the healthy children polyclinic in the Department of Pediatrics were selected for the control group. These children were included in the study after confirming that they did not have any acute or chronic diseases (according to their medical records) and that they did not have any psychiatric disorders (a detailed psychiatric examination was performed by two child and adolescent psychiatrists). Written consent was obtained from the parents of children to participate in the study. 

### 2.2. Search strategy

Laboratory measurements were performed in the Department of Biochemistry. After an overnight (12 h) fast, blood samples were collected from control and patient groups into tubes without anticoagulants using the venipuncture technique. The samples were enabled to clot for 30 min and then centrifuged at 2000 rpm for 10 min as usual. Hemolyzed and lipemic serums were excluded. The aliquoted serum samples were stored at –80 °C for the measurement of Tau and p-Tau_181_ concentrations.

Serum Tau protein and p-Tau181 concentrations were measured by enzyme-linked immunosorbent assay (ELISA) kits, Sunred and Elabscience, respectively. These kits were based on biotin double antibody sandwich technology to determine human Tau and phospho Tau levels in serum, plasma, CSF, urine, and other body fluids; measurements were performed as per the manufacturer’s instructions. The absorbance of each well was measured under 450 nm wavelength. According to the standard concentrations and their corresponding absorbances, we determined the Tau protein and phospho Tau concentrations by calculating the standard curve nonlinear regression equation.

### 2.3. Statistical analysis

Statistical analysis was performed using the TURCOSA (Turcosa Analitik Ltd Co, Turkey) software. The normality of the data was evaluated using the Shapiro–Wilk normality test and Q–Q graphs. Data were expressed as the number for categorical variables and mean ± SD or median (25th–75th percentile) for continuous variables. Age comparison between the two groups was performed using the Independent Samples t-test. In terms of Tau and phospho Tau, comparisons between groups were performed with the Mann–Whitney U test. The relationship between Tau and p-Tau was evaluated with the Spearman correlation analysis. A *p*-value less than 0.05 was considered statistically significant.

### 2.4. Ethical statement

The ethical and methodological aspects of this investigation were approved by the Institutional Review Board of Erciyes University Medical Faculty. Written informed consent was provided by the participants to join this study. We confirm that all the methods were performed in compliance with the relevant guidelines and regulations (Project No: TTU-2017-7506).

## 3. Results

Our study involved 26 male children with ADHD and 26 male controls. The mean age of the controls was 9.08 ± 1.92 and the mean age of the children with ADHD was 8.85 ± 1.89 (*p* = 0.664). 

Total Tau and p-Tau_181_ levels were not normally distributed. Total Tau levels of ADHD were not significantly different between groups (*p* = 0.092). p-Tau_181_ levels in children with ADHD were found to be statistically significantly higher than those in controls (*p* = 0.046) (Table, Figure).

**Table:** Total Tau and p-Tau181 protein levels in patient and control groups.

	Groups		p-value
	Patient (n = 26)	Control (n = 26)	
Total Tau (ng/L)	21.3 (12.4–31.2)	25.4 (23.3–29.5)	0.092
p-Tau181 (pg/mL)	1859.6 (1646.6–2051.5)	1625.7 (1386.6–1896.7)	0.046

**Figure 1:**
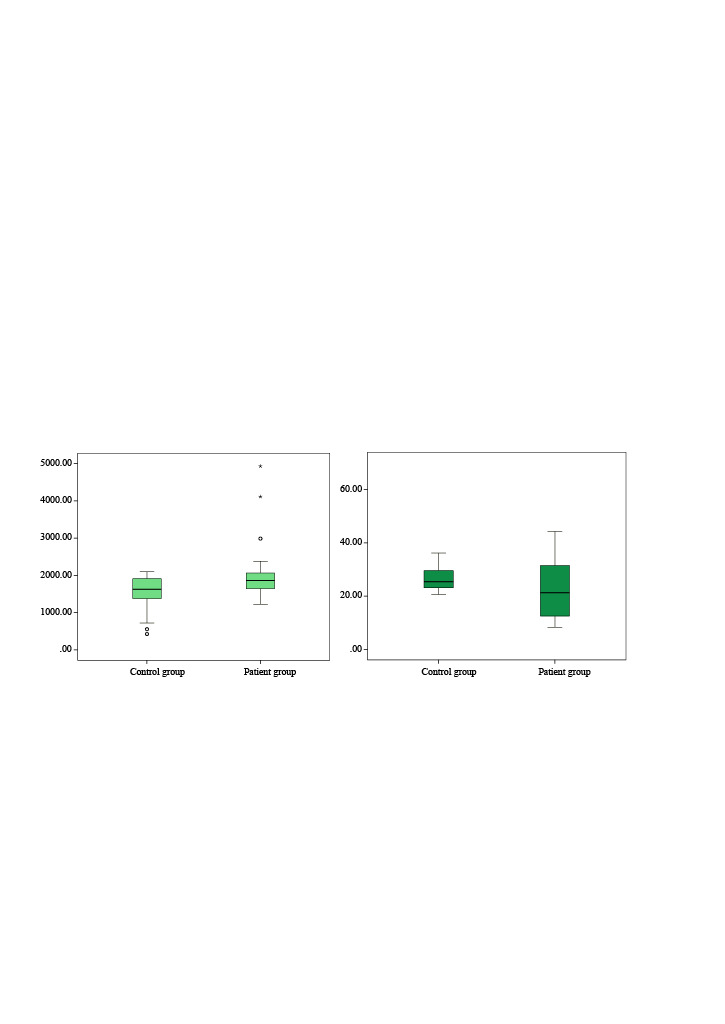
Box plot graphics of p-Tau181 (left) and total Tau (right) in patient and control groups.

In the control group, Tau levels were significantly negatively correlated with p-Tau_181 _levels (r = –0.435 p = 0.026), while this correlation was not found in the ADHD group (*p *= 0.584).

## 4. Discussion

Previous studies have suggested that CSF and plasma Tau/p-Tau_181_ levels can be used as a marker in the diagnosis of some diseases and in determining the severity of axonal damage [6–8]. Although plasma/CSF levels have been evaluated in many neurological diseases, there are limited studies in psychiatric disorders. 

As we mentioned above, there is a study on ADHD in the literature. In this study, only serum total Tau levels were evaluated, not pTau, and were found to be high in the patient group. This finding was interpreted as that ADHD may share a common mechanism with other diseases in terms of tau pathology and may indicate a disturbance in microtubule transport in the brain in this disorder [13].

In this study, we found no statistically significant difference in Tau levels ​​between the patient and control groups. However, the numerical differences in Tau levels (lower in ADHD) suggest that there may be a relationship between Tau and ADHD by an as yet unknown mechanism. A protein produced in the central nervous system can also be detected in serum, but its concentration is approximately 10-fold lower compared to that in CSF. One explanation for this condition is that it leaks into the circulation due to the protein gradient through the impaired blood-brain barrier (BBB) [14]. The low serum Tau levels in our study may be due to the small amount of penetration of this protein through the BBB into the serum. On the other hand, Tau levels may be positively correlated with the number of intact neurons. Tau concentration may vary depending on the dynamic balance between CSF and serum leakage and clearance. Therefore, less disabled patients with a larger number of intact neurons secrete more Tau than patients with pronounced brain atrophy. The numerical higher Tau levels in the control group may be due to this reason. 

To validate these numerical results, these preliminary findings should be confirmed in an independent and large group of ADHD. 

We found that serum p-Tau_181_ levels were significantly higher in the patient group compared to the controls. While there was no correlation between Tau and p-Tau_181_ levels in the patient group, we found a negative correlation in the control group.

It is known that p-Tau_181_ protein reflects neuronal damage and leaks into CSF ​​and serum after axonal damage [14]. The increase in p-Tau_181_ concentration in ADHD can be explained by axonal damage. It is possible for Tau protein to undergo abnormally increased phosphorylation in ADHD, as it has been detected in neurological diseases. The results of our study appear to support that microtubule pathology may play a role in ADHD pathogenesis. 

However, the negative correlation between serum Tau and p-Tau_181_ in the control group can be interpreted as that the biochemistry of Tau may be more complex, and that the level of Tau attempts to balance changes in p-Tau_181_ levels within certain limits.

As it was mentioned above, identifying protein markers in blood has also a few benefits over CSF as it is easily obtained from children patients in the clinic. Therefore, the concept of blood-based biomarkers for ADHD is attractive and can be put into use in many areas, including screening, diagnosis, risk assessment, and supporting drug development in clinical trials. Until now, these two proteins have been evaluated mainly as CSF-based markers in clinical studies [15–19]. These results provide valuable information that serum p-Tau_181_ is a promising potential serum biomarker for the detection of ADHD pathology. Validation of the data obtained with this small-scale study will be possible by supporting larger-scale and well-controlled studies.

There are a few restrictions on this work. Firstly, the patient number was low; therefore, there was not enough statistical force in the investigation. Secondly, the subjects consisted of only male children, female children were not evaluated. Thirdly, kinetic serum Tau and p-Tau_181_ levels were not explored. The uncovered ranges in the serum Tau and p-Tau_181_ protein level could contain kinetic alterations among different patients. Nonetheless, this work shows the elementary proof that the serum p-Tau_181 _level could operate as a supplementary marker to support the early diagnosis of ADHD and the proper estimation of the consequence of ADHD. Importantly, due to the lack of equidistant studies in the literature, it is hard to measure or note the similarities or dissimilarities of our study.

In conclusion, the serum p-Tau_181_ had a natural aptitude or skill for separation between patients and controls from ADHD. Therefore, serum p-Tau_181_ may serve as a predictive protein marker for ADHD patients and may also be used as a prognostic marker during follow-up of patients in the future. 

## Informed consent

The ethical and methodological aspects of this investigation were approved by the Institutional Review Board of Erciyes University Faculty of Medicine. Written informed agreements were supplied by the subscribers to participate in this study.
